# An Assessment of the Effectiveness of Preoperative İmaging Modalities (MRI, CT, and 18F-FDG PET/CT) in Determining the Extent of Disease Spread in Epithelial Ovarian–Tubal–Peritoneal Cancer (EOC)

**DOI:** 10.3390/medicina61020199

**Published:** 2025-01-23

**Authors:** Hülya Kandemir, Hamdullah Sözen, Merve Gülbiz Kartal, Zeynep Gözde Özkan, Samet Topuz, Mehmet Yavuz Salihoğlu

**Affiliations:** 1 Department of Obstetric and Gynecology, Şanlıurfa Training and Research Hospital, 63250 Şanlıurfa, Turkey; 2 Department of Gyneacological Oncology, Istanbul Faculty of Medicine, Istanbul University, 34093 Istanbul, Turkey; drhsozen@gmail.com (H.S.); samettopuz@yahoo.com (S.T.); mehmet.salihoglu@istanbul.edu.tr (M.Y.S.); 3 Department of Radiology, Istanbul Faculty of Medicine, Istanbul University, 34093 Istanbul, Turkey; gulbizkartal@gmail.com; 4 Department of Nuclear Medicine, Istanbul Faculty of Medicine, Istanbul University, 34093 Istanbul, Turkey; zgozdeozkan@yahoo.com

**Keywords:** computed tomography, epithelial ovarian–tubal–peritoneal cancer, magnetic resonance imaging, PET/CT

## Abstract

*Background and Objectives*: Epithelial ovarian–tubal–peritoneal cancer (EOC) is the most common type of ovarian cancer. Optimal cytoreductive surgery is the most important prognostic factor in its management. When complete cytoreduction is anticipated to be challenging, neoadjuvant systemic chemotherapy (NACT) becomes an alternative. Imaging modalities are utilized in the decision-making process for primary treatment. The purpose of this study is to evaluate the diagnostic performance and accuracy of preoperative MRI, CT, and 18F-FDG PET/CT in detecting the extent of EOC. *Materials and Methods*: Between 2017 and 2018, 24 patients with primary (with or without neoadjuvant chemotherapy) or recurrent EOC diagnosed at the Department of Gynecologic Oncology, Istanbul University, Istanbul Faculty of Medicine, were enrolled in this study. These 24 women underwent preoperative imaging modalities within 7 days prior to surgery. The results were compared with histopathological findings, considered the gold standard. *Results*: We evaluated 24 anatomic regions most commonly involved in EOC. The sensitivity of MRI, CT, and PET/CT in detecting ≥ 0.5 cm implants was 95%, 84%, and 86%, respectively. However, when including implants < 0.5 cm, sensitivity decreased significantly to 40%, 38%, and 42%, respectively. The calculated area under the curve (AUC) for tumors, including those < 0.5 cm, was evaluated as weak for all three modalities (MRI: 0.689, CT: 0.678, PET/CT: 0.691), with PET/CT detecting the largest area. For detecting tumors ≥ 0.5 cm, the AUCs were 0.974, 0.921, and 0.923 for MRI, CT, and PET/CT, respectively. The largest AUC was calculated with MRI, and the AUCs for all three methods were evaluated as excellent. Accuracy was comparable among all three imaging modalities, and no statistically significant differences were found (*p* < 0.05). *Conclusions*: While imaging modalities are valuable tools for evaluating abdominal spread in epithelial ovarian cancer (EOC), they have demonstrated limited success in detecting miliary disease. The risk of false negatives for miliary tumors on PET/CT may be mitigated by combining it with other imaging modalities such as MRI or CT. Further investigations are necessary to identify more accurate imaging techniques for this challenging clinical scenario.

## 1. Introduction

Approximately 90% of ovarian cancers arise from the coelomic epithelium or modified mesothelium [1]. Surface epithelial tumors are the most common type of ovarian cancer [2]. Epithelial ovarian, tubal, and peritoneal cancers have been recognized as one neoplastic entity because a lot of evidence points to a common Müllerian epithelium derivation and similar management of these three neoplasms [3,4].

Epithelial ovarian cancer generally presents at an advanced stage (75%), often due to its asymptomatic nature [5]. It remains the leading cause of gynecological cancer mortality, accounting for 43% of deaths [6].

Platinum-based chemotherapy following primary cytoreductive surgery forms the cornerstone of epithelial ovarian cancer treatment [7]. Evidence suggests that optimal cytoreduction is the most important prognostic factor of survival [8,9].

Cytoreductive surgery is more feasible in patients with early-stage disease; however, individuals with advanced-stage EOC frequently present with extensive metastatic disease. This makes primary cytoreductive surgery an aggressive procedure with potentially high perioperative morbidity and mortality.

Patients with advanced-stage disease, extensive tumor burden, older age, poor performance status or medical comorbidities may benefit from neoadjuvant systemic chemotherapy (NACT) [10,11].

Imaging modalities such as magnetic resonance imaging (MRI), computed tomography (CT), and 18-Florodeoksiglukoz positron emission tomography/computed tomography (PET/CT) may provide valuable information regarding the extent of the disease, the location of peritoneal metastases, and the feasibility of complete cytoreductive surgery. This critical information aids gynecologic oncologists in determining the most appropriate therapeutic strategy [12].

Although CT is most commonly used to stage ovarian cancer patients, MRI and PET/CT are increasingly used in specialized centers.

This study aims to assess the sensitivity, specificity, and diagnostic accuracy of these imaging modalities for EOC. Consequently, it seeks to identify which imaging modality is more effective in demonstrating tumor burden and infiltrated areas in regions where EOC most commonly disseminates. This will assist clinicians in selecting an appropriate imaging modality to guide primary treatment decisions, thereby improving surgical planning and reducing postoperative complications.

## 2. Materials and Methods

### 2.1. Patient Selection and Data Collection

This study was conducted at the Department of Obstetrics and Gynecologic Oncology, Our University School of Medicine, between January 2017 and April 2018. Patients with primary (with or without NACT) or recurrent ovarian–fallopian–peritoneal cancer, diagnosed based on clinical, radiological, and biochemical criteria, including physical examination, ultrasound, ascites, elevated CA-125 levels, and cytological analysis of ascitic fluid, were included in this clinical trial.

This study was approved by the Local Ethics Committee on 22 December 2017 (approval number 1537). All participants provided written informed consent after receiving detailed information about the study, in accordance with the Declaration of Helsinki.

Initially, thirty-five patients were recruited, excluding those with contrast media allergies, renal insufficiency, or contraindications to MRI. Eleven patients were excluded: three had non-epithelial cancer, and eight were unable to undergo surgery due to a lack of available intensive care unit beds. Twenty-four patients diagnosed with EOC who met the inclusion criteria underwent imaging modalities (MRI, CT, and PET/CT) within 7 days prior to surgery. Surgical specimens were histopathologically examined and served as the gold standard.

### 2.2. Surgical Criteria

All surgical procedures in this study were conducted by the same experienced team of gynecologic oncologists. The surgeons were aware of the preoperative imaging findings from MRI, CT, and PET/CT scans. All surgical procedures aimed at maximal cytoreduction, involving pelvic and para-aortic lymphadenectomy accessed through a median laparotomy incision. A comprehensive assessment was performed targeting 24 distinct anatomic regions frequently implicated in the metastatic spread of ovarian cancer, including the bladder peritoneum, Douglas’ pouch, sigmoid colon/rectal serosa, right paracolic gutter, left paracolic gutter, small bowel mesentery, small bowel serosa, transverse colon serosa, colon mesentery, splenic capsule, greater omentum, omental cake, celiac trunk, Morrison’s pouch, porta hepatis, liver surface, right and left diaphragms, pelvic and common iliac lymph nodes, paracaval lymph nodes, interaortacaval lymph nodes, left para-aortic lymph nodes, lesser omentum, and liver parenchyma, were specifically evaluated. Tumor extent was determined by a consensus of two operators. During surgical exploration, the presence or absence of metastasis, implants, and thickening in these areas was noted, along with their dimensions if present. Staging was performed according to the 2014 FIGO staging system [13].

### 2.3. Histopathologic Evaluation

All specimens were evaluated by two experienced gynecologic pathologists from the Department of Pathology, Istanbul University, Istanbul Faculty of Medicine. The classification was based on the 2014 World Health Organization (WHO) classification of tumors [14]. In cases of discrepancy between surgical specimens and biopsy results, the histopathologic findings were considered the gold standard.

### 2.4. Imaging Analysis

MRI, CT, and 18F-FDG PET/CT images were obtained using standardized protocols and equipment to minimize variability. Details are provided in Supplementary S1. The acquired images were uploaded to the PACS (Picture Archiving and Communication Systems) and stored in the hospital database. CT and MRI images were independently evaluated by a radiologist with 13 years of experience in gynecologic oncology, while PET/CT images were interpreted by nuclear medicine physicians with 12 years of experience. Both the radiologist and the nuclear medicine physician were blinded to the patients’ clinical data, laboratory results, and histopathologic findings. When interpreting the images, both experts systematically assessed 24 pre-defined anatomical regions commonly involved in ovarian cancer metastasis for the presence or absence of metastases, implants, or thickening, noting their size and FDG uptake. Histopathological findings served as the gold standard. A template table for recording the assessment findings for each patient is provided in Supplementary S2.

### 2.5. Statistical Analysis

Data were analyzed using the IBM Statistical Package for the Social Sciences (SPSS)-21. Histopathology served as the gold standard. To evaluate the sensitivity, specificity, positive predictive value (PPV), negative predictive value (NPV), and accuracy of each imaging modality in detecting tumors in 24 predefined anatomical regions commonly involved in ovarian cancer, 2 × 2 contingency tables were created. The results from these tables were entered into formulas in Microsoft Excel 2016 to calculate percentages. Additionally, receiver operating characteristic (ROC) curve analysis was performed to calculate the area under the curve (AUC) for each imaging modality. Pairwise comparisons of ROC curves for CT, MRI, and PET/CT were performed using the DeLong test with the MedCalc software (v23.1.3). A *p*-value < 0.05 was considered statistically significant for these comparisons. All analyses were conducted with a 95% confidence interval, and statistical significance was set at *p* < 0.05.

## 3. Results

A total of 24 patients were included in the study, and their demographic data are presented in Table 1.

The diagnostic performance of CT, MRI, and PET/CT, even for tumors smaller than 0.5 cm, is presented in Table 2, with histopathology serving as the gold standard.

**Figure 1:**
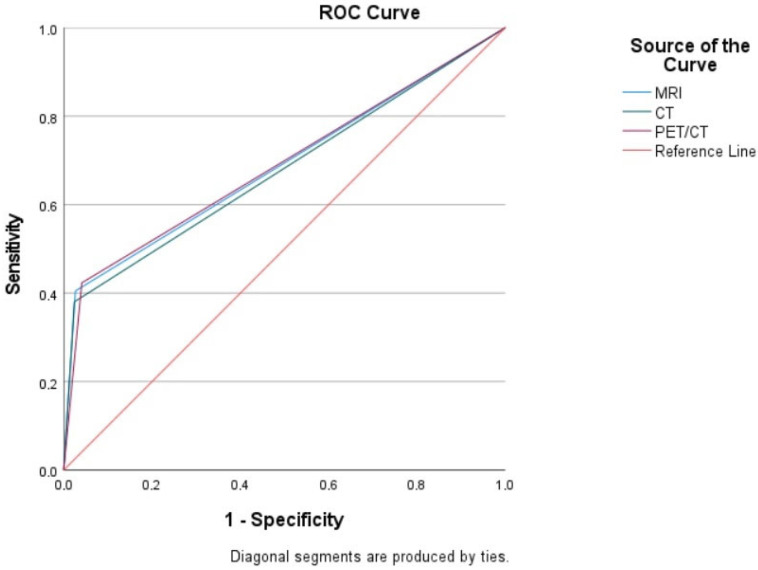
The sROC curves demonstrating the performance of CT, MRI, and PET/CT when including tumors smaller than 0.5 cm.

Figure 1 presents the sROC curves, demonstrating the performance of CT, MRI, and PET/CT when including tumors smaller than 0.5 cm. While not statistically significant, PET/CT demonstrated a slightly higher specificity and AUC (0.691) compared to MRI (0.689) and CT (0.678). For lesions smaller than 0.5 cm, pairwise comparisons of the ROC curves using the DeLong test showed no statistically significant differences between the AUC values of PET/CT (0.691), MRI (0.689), and CT (0.678) (CT vs. MRI: *p* = 0.4206; CT vs. PET/CT: *p* = 0.4570; MRI vs. PET/CT: *p* = 0.9023). Given that the AUC values for all three imaging modalities ranged between 0.60 and 0.70, it was concluded that they exhibited poor sensitivity in detecting tumors smaller than 0.5 cm, as shown in Table 3.

The diagnostic performance of CT, MRI, and PET/CT, for tumors equal to or larger than 0.5 cm, is presented in Table 4, with histopathology serving as the gold standard.

**Figure 2:**
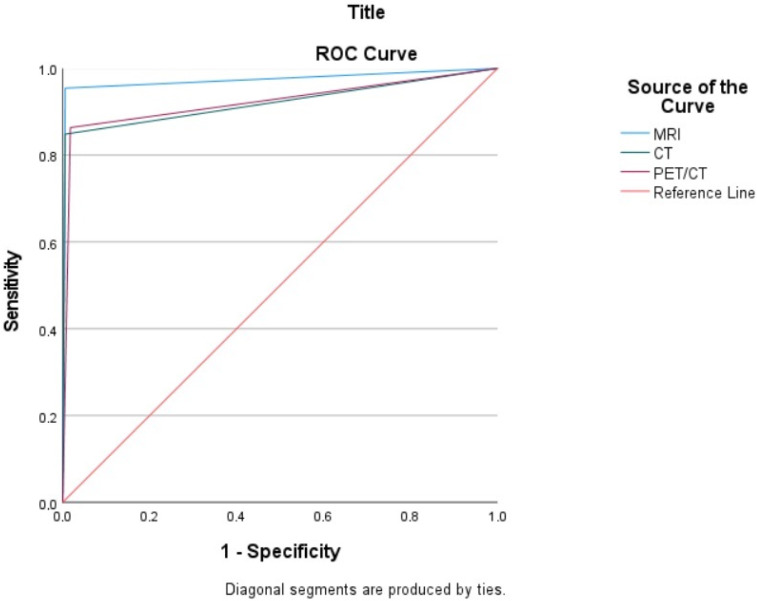
The sROC curves demonstrating the performance of CT, MRI, and PET/CT exclusively for tumors with a diameter of 0.5 cm or greater.

Figure 2 presents the sROC curves, demonstrating the performance of CT, MRI, and PET/CT exclusively for tumors with a diameter of 0.5 cm or greater. MRI demonstrated the highest diagnostic accuracy, with an AUC of 0.974. PET/CT and CT yielded AUC values of 0.923 and 0.921, respectively. Pairwise comparisons of ROC curves, performed using the DeLong test, revealed that MRI had statistically significantly higher AUC values than both CT (*p* = 0.0059) and PET/CT (*p* = 0.0304). No statistically significant difference was found between the AUC values of CT and PET/CT (*p* = 0.9470). The consistently high AUC values (0.90–0.99) across all three imaging modalities indicate a significantly high sensitivity for detecting tumors measuring 0.5 cm or larger, as shown in Table 5.

## 4. Discussion

Advanced epithelial ovarian cancer is commonly managed with a combination of cytoreductive surgery followed by chemotherapy [7]. However, patients who are not surgical candidates, often due to poor performance status or unresectable disease, may be considered for NACT.

Accurate identification of the primary tumor and metastases is crucial for selecting the most appropriate therapeutic strategy and predicting the feasibility of optimal resection [15]. Laparoscopy provides direct visualization of abdominal organs, facilitating accurate topographic mapping of the tumor with a low false-negative rate of 3% [16]. Nevertheless, it is an invasive procedure that requires general anesthesia [17]. Imaging modalities such as CT, MRI, and PET/CT can provide detailed information regarding tumor extent and the location of peritoneal metastases, which is crucial for staging, primary treatment selection, assessment of postoperative complications, and survival prediction [18,19].

CT is the most commonly used initial imaging modality for staging and follow-up of ovarian cancer worldwide [20]. It offers several advantages such as a short acquisition time, high image quality, and low cost [21].

A prospective study demonstrated that contrast-enhanced multidetector CT has a sensitivity ranging from 25–100% and a specificity of 78–100% in detecting peritoneal dissemination. The sensitivity of CT was significantly reduced for implants smaller than 0.5 cm, ranging from 11% to 48% [22].

Based on our findings, CT exhibited a sensitivity of 38% and a specificity of 97.5% in detecting tumor implants, including those smaller than 0.5 cm, in areas frequently involved in EOC. In contrast, when we limited our analysis to tumor implants measuring ≥ 0.5 cm, the overall sensitivity increased to 84.8%, and the specificity reduced to 94.4%.

Most studies agree that CT sensitivity increases with the size of implants [23]. In fact, CT sensitivity has been found to be 28% for tumors smaller than 0.5 cm, 72% for those between 0.5 and 5 cm, and 90% for those larger than 5 cm [23].

MRI is an expensive imaging technique that is time-consuming, susceptible to motion artifacts, and has many contraindications. The addition of diffusion-weighted MRI sequences has enhanced the sensitivity of MRI in detecting peritoneal dissemination [24] [25]. The use of fat-suppressed, gadolinium-enhanced, and diffusion-weighted images has been shown to yield higher sensitivity than CT [26,27].

Our study included both diffusion-weighted and gadolinium-enhanced imaging. According to our data, the overall sensitivity of MRI was found to be 40.4%, and the specificity was 97.3% in detecting tumor implants, including those smaller than 0.5 cm, in areas frequently involved in EOC. Our findings revealed that MRI had a higher overall sensitivity (95.4%) and specificity (99.4%) in detecting tumor implants ≥ 0.5 cm. Nevertheless, for miliary implants, both CT and MRI exhibited limited performance, with no significant superiority of one modality over the other.

Low et al. reported a sensitivity of 84% for gadolinium-enhanced MRI and 54% for CT in detecting tumors of all sizes. For miliary tumor spread (<0.5 cm), they found a sensitivity of 75–80% for gadolinium-enhanced MRI and 22–33% for CT [28]. As demonstrated, imaging techniques such as MRI and CT may fail to detect tumor infiltration of abdominal surfaces or extrapelvic spread, thus limiting their ability to accurately stage the disease [29]. Other imaging modalities might be considered in these circumstances.

However, PET/CT has several drawbacks, including decreased resolution in imaging miliary carcinomas, image artifacts due to patient bowel movements and respiration, artifacts caused by excretion in the bladder and gastrointestinal system, radiation exposure, and high cost [30].

In the regions we examined, PET/CT demonstrated an overall sensitivity of 86.3% and specificity of 98% in detecting tumor implants ≥ 0.5 cm. However, when we included miliary (<0.5 cm) tumor implants, the sensitivity decreased to 42% while the specificity remained relatively high at 95%.

PET/CT has been shown to have high diagnostic accuracy for tumor masses larger than 0.5 cm in advanced-stage ovarian cancer. However, its ability to detect smaller tumors, those less than 0.5 cm, is limited, with a sensitivity of 78.9% and specificity of 68.4% [15]. There was no significant difference in FDG uptake between regions without tumor and those with tumor nodules smaller than 0.5 cm.

A study comparing diffusion-weighted MRI and PET/CT for the detection of peritoneal cancer reported sensitivities of 74% and 63%, respectively. For subcentimeter tumors (<1 cm), sensitivities decreased to 42% and 50% for MRI and PET/CT, respectively. These results indicate that both imaging techniques may have limitations in detecting pelvic, mesenteric carcinomatosis, and miliary tumors [31].

The whole-body coverage of PET/CT is an advantage, as it can detect extra-abdominal metastases. In our study, PET/CT raised suspicion of metastases in the cardiophrenic lymph nodes of two patients, which was subsequently confirmed by histopathology following lymphadenectomy. Of the 24 regions evaluated in 24 patients, 17 areas initially diagnosed as metastatic on PET/CT were not confirmed histologically. This discrepancy can be explained by the known tendency of PET/CT to yield false-positive results due to physiologic and inflammatory processes or reactive lymph nodes [32]. Such overestimations of disease extent can lead to unnecessary surgical and chemotherapy treatments.

A comparison of sensitivities among CT (88%), MRI (94%), and PET/CT (94%) in previous studies revealed no statistically significant difference between MRI and PET/CT [33]. Considering these findings, MRI, with its lower cost and greater accessibility, may be the preferred initial imaging modality [34]. A separate study evaluating the sensitivities of CT, MRI, and PET/CT in diagnosing peritoneal carcinomatosis reported sensitivities of 96%, 98%, and 95%, respectively, and specificities of 92%, 84%, and 96%. While MRI exhibited the highest sensitivity and PET/CT the highest specificity, no statistically significant differences were observed among the three techniques. Due to its accessibility, shorter acquisition time, and lower cost, CT was recommended as the initial imaging modality. Nevertheless, if CT results were inconclusive or negative, MRI and PET/CT could provide complementary information [35].

In the diagnosis and treatment planning of ovarian cancer, MRI and CT are utilized to assess tumor burden, localization, and the feasibility of complete resection. CT is rapid, cost-effective, and widely accessible. Notably, Whole-Body Diffusion-Weighted Imaging (WB-DWI) MRI demonstrates superior efficacy in characterizing tumor features and detecting peritoneal dissemination [18]. While PET/CT is as effective as CT in determining the extent of disease spread, it exhibits a slight advantage in identifying lymph node and distant metastases. In cases where CT and MRI results are negative but there is a suspicion of recurrence, the use of PET/CT may be considered [36]. The combined use of PET/CT, CT, and MRI increases the overall cost. The results of similar studies evaluating the effectiveness of imaging modalities in the diagnosis of ovarian cancer are summarized in Table 6.

### 4.1. Limitations

1. The small sample size may have limited the generalizability of the findings.
2. The single-center design may have limited the generalizability of our findings.

### 4.2. Strenghts

1. This study involved a homogenous study population consisting exclusively of EOC patients.
2. This study had a prospective study design.
3. Histopathological confirmation was used as the gold standard.
4. For each imaging modality (CT, MRI, and PET/CT), standard institutional protocols were adhered to.

To increase the impact and generalizability of future research, larger sample sizes and multi-center collaborations are recommended.

## 5. Conclusions

While tumors ≥ 0.5 cm are often characterized with greater specificity by MRI, CT, and PET/CT, advanced-stage epithelial ovarian, tubal, and peritoneal cancers frequently exhibit miliary spread. Therefore, to assess miliary spread, we included tumors < 0.5 cm in our analysis. The sensitivities of MRI, CT, and PET/CT were 40%, 38%, and 42%, respectively, suggesting insufficient performance. The combined use of all three modalities did not improve diagnostic accuracy. FDG-PET/CT was not more accurate than CT and MRI for detecting intra-abdominal disease spread and did not offer additional clinical value for preoperative treatment planning in this study. Although preoperative imaging is valuable for assessing disease dissemination, a reliable non-invasive method to assess operability is still lacking. New imaging modalities, such as PET/MRI, could be investigated in epithelial ovarian cancer.

## Figures and Tables

**Table 1:** The demographic and clinical characteristics of the patients.

Characteristics	n: 24
Age (Year), mean ± SD (Min–Max)	53.5 ± 12.3 (27–79)
Primary cancer, n (%)	16 (66.7)
Recurrent cancer, n (%)	8 (33.3)
FIGO stage	
Stage 1, n (%)	2 (8.3)
Stage 2, n (%)	1 (4.2)
Stage 3, n (%)	21 (87.5)
Histology	
Serous, n (%)	21 (87.5)
Mucinous, n (%)	1 (4.16)
Clear cell, n (%)	1 (4.16)
Endometriod, n (%)	1 (4.16)

Abbreviations: SD, standard deviation; n, number of patients; FIGO, International Federation of Gynecology and Obstetrics, Min, minimum; Max, maximum.

**Table 2:** Diagnostic performance of CT, MRI, and PET/CT, including tumors < 0.5 cm in size (histopathology as gold standard).

Imaging Modalities	Sensitivity % (95% CI)	Specificity % (95% CI)	PPV %	NPV %	Accuracy %
CT Overall	38.04 (36.32–39.74)	97.58 (97.23–97.91)	86.11	79.96	81.0
MRI Overall	40.49 (38.74–42.23)	97.34 (96.97–97.69)	85.71	80.56	81.0
PET/CT Overall	42.33 (40.58–44.08)	95.88 (95.44–96.32)	80.23	80.82	81.0

Abbreviations: CT, computed tomography; MRI, magnetic resonance imaging; PET/CT, positron emission tomography; PPV, positive predictive value; NPV, negative predictive value; CI, confidence interval.

**Table 3:** Area under the curve (AUC) for all tumor sizes (including < 0.5 cm).

Test Result Variable(s)	Area	Std. Error ^a^	Asymptotic Sig. ^b^	Asymptotic 95% Confidence Interval	
				Lower Bound	Upper Bound
MRI	0.689	0.027	0.000	0.636	0.743
CT	0.678	0.027	0.000	0.624	0.732
PET/CT	0.691	0.027	0.000	0.638	0.744

The test result variable(s): MRI, CT, and PET/CT have at least one tie between the positive actual state group and the negative actual state group. Statistics may be biased. ^a^ Under the nonparametric assumption. ^b^ Null hypothesis: true area = 0.5. CT, computed tomography; MRI, magnetic resonance imaging; PET/CT, positron emission tomography.

**Table 4:** Diagnostic performance of CT, MRI, and PET/CT in tumors ≥ 0.5 cm (histopathology as gold standard).

Imaging Modalities	Sensitivity % (95% CI)	Specificity % (95% CI)	PPV %	NPV %	Accuracy %
CT Overall	84.85 (84.51–85.18)	99.41 (99.38–99.43)	94.92	98.07	98.0
MRI Overall	95.45 (95.37–95.53)	99.41 (99.40–99.42)	95.45	99.41	99.0
PET/CT Overall	86.36 (85.92–86.80)	98.04 (97.76–98.32)	85.07	98.23	97.0

Abbreviations: CT, computed tomography; MRI, magnetic resonance imaging; PET/CT, positron emission tomography; PPV, positive predictive value; NPV, negative predictive value; CI, confidence interval.

**Table 5:** Area under the curve (AUC) for tumors ≥ 0.5 cm.

Test Result Variable(s)	Area	Std. Error ^a^	Asymptotic Sig. ^b^	Asymptotic 95% Confidence Interval	
				Lower Bound	Upper Boun
MRI	0.974	0.015	0.000	0.945	1.000
CT	0.921	0.026	0.000	0.870	0.973
PET/CT	0.923	0.025	0.000	0.874	0.972

The test result variable(s): MRI, CT, and PET/CT have at least one tie between the positive actual state group and the negative actual state group. Statistics may be biased. ^a^ Under the nonparametric assumption. ^b^ Null hypothesis: true area = 0.5. CT, computed tomography; MRI, magnetic resonance imaging; PET/CT, positron emission tomography.

**Table 6:** Ovarian cancer imaging: diagnostic performance from similar studies.

Study	Imaging Modalities	Sensitivity	Specificity	Key Findings
Gu, P. et al. A systematic review and meta-analysis 2009 [36]	CT MRI PET/CT	79% 75% 91%	84% 78% 88%	PET/CT may complement current surveillance methods, especially in patients with elevated CA 125 and negative CT or MRI
Yuan et al. A meta-analysis 2012 [37]	CT MRI PET/CT	42.6% 54.7% 73.2%	95.0% 88.3% 96.7%	PET or PET/CT is more accurate than CT and MRI in the detection of lymph node metastasis in patients with ovarian cancer.
Michielsen et al., 2014 [18]	CT WB-DWI/MRI PET/CT	65% 91% 52%	82% 91% 85%	WB-DWI/MRI shows high accuracy in characterizing primary tumors, peritoneal tumors, and distant staging.
Schmidt et al., 2015 [35]	CT MRI PET/CT	96% 98% 95%	92% 84% 96%	MRI had the highest sensitivity, and FDG PET/CT had the highest specificity, with no significant differences between the three techniques. CT is the preferred choice for stand-alone examination due to its speed, cost-effectiveness, and wide availability.
Van’t Sant et al. A meta-analysis 2020 [38]	CT MRI PET/CT	68% 92% 80%	88% 85% 90%	DW-MRI and PET/CT showed comparable diagnostic performance.
Tisili et al. A systematic review and meta-analysis 2024 [39]	CT MRI PET/CT	79.7% 82.7% 93.7%	92.1% 90.3% 91.5%	Both FDG PET/CT and MRI have comparably higher per-patient diagnostic accuracy.

Abbreviations: DWI: diffusion-weighted imaging; MRI: magnetic resonance imaging; PET: positron emission tomography; CT: computerized tomography; WB: whole-body; MD: multi dedector; CA: cancer antigen; CI: confidence interval.

## Data Availability

The data and materials used in this study are available upon request. For access to the data or materials, please contact the corresponding author.

## Supplementary Materials

The following supporting information can be downloaded at: https://www.mdpi.com/article/10.3390/medicina61020199/s1. This includes Supplementary S1: Imaging (MRI, CT, and 18F-FDG PET/CT) acquisition parameters and protocols; Supplementary S2: comparative evaluation table: imaging, surgical, and histopathological findings.

## Abbreviations

The following abbreviations are used in this manuscript:

AUC	Area under the curve
CA-125	Cancer Antigen 125
CI	Confidence interval
CT	Computed tomography
EOC	Epithelial ovarian–tubal–peritoneal cancer
FDG	Fluorodeoxyglucose
FIGO	International Federation of Gynecology and Obstetrics
MRI	Magnetic resonance imaging
NACT	Neoadjuvant chemotherapy
NPV	Negative predictive value
PET/CT	Positron emission tomography/computed tomography
PPV	Positive predictive value
sROC	Summary receiver operating characteristic
WB-DWI	Whole-Body Diffusion-Weighted Imaging
