# Supplementary material for: An Assessment of the Effectiveness of Preoperative İmaging Modalities (MRI, CT, and 18F-FDG PET/CT) in Determining the Extent of Disease Spread in Epithelial Ovarian–Tubal–Peritoneal Cancer (EOC)
Source: Medicina (Kaunas). 2025 Jan 23;61(2):199. doi: 10.3390/medicina61020199 (PMC11857206; doi:10.3390/medicina61020199)
Supplement: Supplementary file 1 [file medicina-61-00199-s001.zip › Supplementary S1.pdf]

S1: Imaging (MRI, CT, And 18F-FDG PET/CT) Acquisition Parameters and Protocols

CT imaging criteria: A 64-detector CT scan (Toshiba Aquilon 64) was performed in the supine position. The patient did not receive oral contrast. Intravenous administration of 90 mL of non-ionic contrast agent was performed at a rate of 2.5 mL per second. Imaging was acquired 30 seconds post-contrast administration. Axial imaging was performed from the diaphragmatic dome to the pelvic floor with 0.5 cm slice thickness, encompassing the entire abdomen and pelvis.

MRI imaging criteria: Images were acquired using a 1.5T imaging system (Philips Achieva, Philips Medical Systems, Best, Hollanda) and gadolinium-based contrast agents. A 16-channel abdominal coil was used. T2-weighted (T2W) turbo spin echo, breath-hold multisection single-shot spin echo, and echo-planar diffusion-weighted imaging (DWI) sequences (with fat suppression to reduce chemical shift artifacts) were acquired. Additionally, breath-hold T1-weighted, fat-saturated spoiled gradient-echo sequences were acquired pre-contrast, dynamically, and in the delayed phase. The imaging parameters for these sequences are presented in Table 8. A gadolinium-based contrast agent was administered intravenously at a dose of 0.1 mmol/kg, followed immediately by image acquisition.

Parameter	T2A TSE*	Multi-section single-shot DWI*	T1 FS sGE***
Slice thickness (mm)	6-8	6	4
Slice Interval (mm)	1,5-2	1	0,8
Repetition Time (TR) (ms)	809	1812	5,11
Echo Time (TE) (ms)	290	868	2,51
NEX****	1	3	1
Flip Angle	90	90	10
Matriks	216*216	216*256	352*352

*TSE: Turbo spin echo; **DWI: Diffusion-weighted imaging; ***FS sGE: Fat-suppressed spoiled gradient echo; ****NEX: Number of excitations

DWI were acquired with b-values of 0, 500, and 1000 s/mm². A reduction factor of 2 and an echo planar imaging factor of 53 were used.

PET/CT imaging criteria: Fluorine-18 fluorodeoxyglucose (F-18 FDG) was injected into patients with blood glucose levels less than 200 mg/dL. A whole-body PET/CT scan was performed using a Siemens Biograph™ 6 TruePoint™ scanner, and standard uptake values (SUVs) were determined. A minimum 6-hour fast was required for all patients prior to radiopharmaceutical injection. Patients taking metformin, an oral antidiabetic medication, were advised to discontinue the medication 72 hours prior to the procedure to prevent any interference from drug-induced bowel activity. To prevent interference with muscle activity uptake, patients using insulin were instructed to withhold rapid-acting insulin for at least 4 hours and other insulin formulations for at least 12 hours prior to the radiopharmaceutical injection. Prior to radiopharmaceutical injection, patients' blood glucose levels were checked. Patients with blood glucose levels below 200 mg/dL received an injection of 0.2 mCi/kg fluorine-18 fluorodeoxyglucose (F-18 FDG). Patients were positioned in semi-recumbent chairs for 60-90 minutes to allow for adequate radiopharmaceutical distribution in tissues and to reduce background activity prior to imaging. Transmission scans were acquired using the 4-slice spiral CT component of

the device with a slice thickness of 4 mm. Emission data were collected using the 3D PET component of the device in 6-8 bed positions, with a 3-minute acquisition time per position, covering the region from the vertex to the mid-thigh. The emission images underwent CT-based attenuation correction. Iterative reconstruction (ordered subset expectation maximization-OSEM) was applied to the PET data using 2 iterations, 8 subsets, and a 0.5 cm filter. The reconstructed PET images, following attenuation correction, were evaluated in conjunction with CT images and fused images. Visual assessment was performed in axial, coronal, and sagittal planes, and using three-dimensional maximum intensity projection (MIP). Follow-up spot images were obtained 1-2 hours after the initial scan in areas of interest that were considered suspicious on the initial image interpretation. Images were initially visually assessed. Areas demonstrating increased uptake on PET images compared to background activity, outside of regions of physiologic uptake, were considered pathological. Corresponding CT images were used for tumor characterization. Regions of interest (ROIs) were drawn over the tumor areas. Standardized uptake values (SUVs), a semi-quantitative measure calculated by dividing the activity within the ROI (mCi/ml) by the injected dose (mCi/kg), were determined.
