# Supplementary material for: An Assessment of the Effectiveness of Preoperative İmaging Modalities (MRI, CT, and 18F-FDG PET/CT) in Determining the Extent of Disease Spread in Epithelial Ovarian–Tubal–Peritoneal Cancer (EOC)
Source: Medicina (Kaunas). 2025 Jan 23;61(2):199. doi: 10.3390/medicina61020199 (PMC11857206; doi:10.3390/medicina61020199)
Supplement: Supplementary file 1 [file medicina-61-00199-s001.zip › Supplementary S2.pdf]

S2: Comparative Evaluation Table: Imaging, Surgical, and Histopathological Findings

Patient Number:

File Number:

	Region	*MRI*	*CT*	*PET/CT*	*Surgical Findings*	*Histopathology Findings*
1	Bladder Peritoneum					
2	Douglas' Pouch					
3	Sigmoid Colon/Rectal Serosa					
4	Right Paracolic Gutter					
5	Left Paracolic Gutter					
6	Small Bowel Mesentery					
7	Small Bowel Serosa					
8	Transverse Colon Serosa					
9	Colon Mesentery					
10	Splenic Capsule					
11	Greater Omentum					

12	Omental Cake
13	Celiac Trunk
14	Morrison's Pouch
15	Porta Hepatis
16	Liver Surface
17	Right Diaphragm
18	Left Diaphragm
19	Pelvic and Common Iliac Lymph Nodes
20	Paracaval Lymph Nodes
21	Interaortacaval Lymph Nodes
22	Left Para-aortic Lymph Nodes
23	Lesser Omentum
24	Liver Parenchyma
